# SARS-Coronavirus Replication Is Supported by a Reticulovesicular Network of Modified Endoplasmic Reticulum

**DOI:** 10.1371/journal.pbio.0060226

**Published:** 2008-09-16

**Authors:** Kèvin Knoops, Marjolein Kikkert, Sjoerd H. E. van den Worm, Jessika C Zevenhoven-Dobbe, Yvonne van der Meer, Abraham J Koster, A. Mieke Mommaas, Eric J Snijder

**Affiliations:** 1 Section Electron Microscopy, Department of Molecular Cell Biology, Leiden University Medical Center, Leiden, The Netherlands; 2 Molecular Virology Laboratory, Department of Medical Microbiology, Leiden University Medical Center, Leiden, The Netherlands; Fred Hutchinson Cancer Research Center, United States of America

## Abstract

Positive-strand RNA viruses, a large group including human pathogens such as SARS-coronavirus (SARS-CoV), replicate in the cytoplasm of infected host cells. Their replication complexes are commonly associated with modified host cell membranes. Membrane structures supporting viral RNA synthesis range from distinct spherular membrane invaginations to more elaborate webs of packed membranes and vesicles. Generally, their ultrastructure, morphogenesis, and exact role in viral replication remain to be defined. Poorly characterized double-membrane vesicles (DMVs) were previously implicated in SARS-CoV RNA synthesis. We have now applied electron tomography of cryofixed infected cells for the three-dimensional imaging of coronavirus-induced membrane alterations at high resolution. Our analysis defines a unique reticulovesicular network of modified endoplasmic reticulum that integrates convoluted membranes, numerous interconnected DMVs (diameter 200–300 nm), and “vesicle packets” apparently arising from DMV merger. The convoluted membranes were most abundantly immunolabeled for viral replicase subunits. However, double-stranded RNA, presumably revealing the site of viral RNA synthesis, mainly localized to the DMV interior. Since we could not discern a connection between DMV interior and cytosol, our analysis raises several questions about the mechanism of DMV formation and the actual site of SARS-CoV RNA synthesis. Our data document the extensive virus-induced reorganization of host cell membranes into a network that is used to organize viral replication and possibly hide replicating RNA from antiviral defense mechanisms. Together with biochemical studies of the viral enzyme complex, our ultrastructural description of this “replication network” will aid to further dissect the early stages of the coronavirus life cycle and its virus-host interactions.

## Author Summary

Viruses with a positive-stranded RNA genome replicate in the cytoplasm of infected host cells. Their replication is driven by a membrane-bound viral enzyme complex that is commonly associated with modified intracellular membranes. Little is understood about the formation and architecture of these replication structures and their exact role in viral RNA synthesis. We used electron microscopy and tomography for the three-dimensional imaging of the membrane alterations induced by severe acute respiratory syndrome (SARS)-coronavirus, a member of the virus group with the largest RNA genome known to date. Previously, coronaviruses were reported to induce large numbers of isolated “double-membrane vesicles” (DMVs). However, our present studies reveal an elaborate reticulovesicular network of modified endoplasmic reticulum membranes with which SARS-coronavirus replicative proteins are associated. The lumen of this unique membrane network contains numerous large (diameter 250–300 nm) “inner vesicles,” which were formerly thought to reside in isolated DMVs. Intriguingly, although the interior of these vesicles does not appear to be connected to the cytosol, it labels abundantly for double-stranded RNA, which presumably is present at the site of viral RNA synthesis. The ultrastructural dissection of this elaborate “replication network” shows how coronaviruses extensively reorganize the host cell's membrane infrastructure, to coordinate their replication cycle, and possibly also hide replicating RNA from antiviral defense mechanisms.

## Abstract 3

Positive-strand RNA virus replication is associated with membranes in the host cell's cytoplasm. Here, advanced 3D electron microscopy reveals that SARS-coronavirus induces an elaborate reticulovesicular network of modified ER membranes that supports viral RNA synthesis.

## Introduction

Viruses rely on the host cell's infrastructure and metabolism during essentially all stages of their replication cycle and have therefore adopted strategies to coordinate a variety of molecular interactions in both time and intracellular space. The fact that the replication complexes of positive-strand RNA (+RNA) viruses of eukaryotes are invariably associated with (modified) intracellular membranes appears to be a striking example of such a strategy [1–8]. Specific +RNA virus replicase subunits are targeted to the membranes of particular cell organelles that are subsequently modified into characteristic structures with which viral RNA synthesis is associated. The morphogenesis, ultrastructure, and function of these complexes, sometimes referred to as “viral factories,” are only beginning to be understood. They may facilitate the concentration of viral macromolecules and provide a membrane-based structural framework for RNA synthesis. Other potential benefits include the possibility to coordinate different steps in the viral life cycle and to delay the induction of host defense mechanisms that can be triggered by the double-stranded RNA (dsRNA) intermediates of +RNA virus replication [2,9,10]. Defining the structure–function relationships that govern the membrane-associated replication of +RNA viruses, a large virus cluster including many important pathogens, will enhance our general understanding of their molecular biology and may have important implications for the development of novel antiviral control strategies.

**Figure 1:**
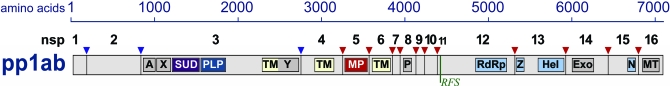
The Coronavirus Replicase Polyprotein The domain organization and proteolytic processing map of the SARS-CoV replicase polyprotein pp1ab. The replicase cleavage products (nsp1–16) are numbered, and conserved domains are highlighted (blue, conserved across nidoviruses; grey, conserved in coronaviruses). These include transmembrane domains (TM), protease domains (PLP and MP), and (putative) RNA primase (P), helicase (HEL), exonuclease (Exo), endoribonuclease (N), and methyl transferase (MT) activities. For more details, see [14,18]. The delineation of amino acids encoded in ORF1a and ORF1b is indicated as RFS (ribosomal frameshift), and arrows represent sites in pp1ab that are cleaved by the nsp3 papain-like protease (in blue) or the nsp5 main protease (in red).

Following the 2003 outbreak of severe acute respiratory syndrome (SARS; for a review, see [11]), the coronavirus family of +RNA viruses received worldwide attention. In addition to SARS-coronavirus (SARS-CoV), several other novel family members were identified, including two that also infect humans [12]. Coronaviruses, and other members of the nidovirus group, have a polycistronic genome and employ various transcriptional and (post)translational mechanisms to regulate its expression [13,14]). The gene encoding the replicase/transcriptase (commonly referred to as “replicase”) comprises about two-thirds of the coronavirus genome, which—at 27–31 kb—is the largest RNA genome known to date. The replicase gene consists of open reading frames (ORFs) 1a and 1b, of which the latter is expressed by a ribosomal frameshift near the 3′ end of ORF1a. Thus, SARS-CoV genome translation yields two polyproteins (pp1a and pp1ab) that are autoproteolytically cleaved into 16 nonstructural proteins (nsp1 to 16; Figure 1) by proteases residing in nsp3 and nsp5 [15–17]. Several of the replicative enzymes of coronaviruses, like an RNA-dependent RNA polymerase (RdRp) and a helicase, are common among +RNA viruses, but they also contain a variety of functions that are rare or absent in other +RNA viruses, including a set of intriguing proteins that are distantly related to cellular RNA processing enzymes [13,14,18]. The complexity of coronavirus RNA synthesis is further highlighted by the fact that it entails not only the production of new genome molecules from full-length negative-strand RNA (“replication”), but also a unique mechanism of discontinuous RNA synthesis to generate subgenome-length negative-strand RNA templates for subgenomic mRNA production (“transcription”) [19,20]. The resulting set of subgenomic transcripts (eight in the case of SARS-CoV) serves to express structural and accessory protein genes in the 3′-proximal domain of the genome. Ultimately, new coronavirions are assembled by budding of nucleocapsids into the lumen of pre-Golgi membrane compartments [21,22].

The nidovirus replicase includes several (presumed) multispanning transmembrane proteins that are thought to physically anchor the replication/transcription complex (RTC) to intracellular membranes. In the case of coronaviruses, these domains reside in nsp3, nsp4, and nsp6 (Figure 1) [23,24]. In the cytoplasm of infected cells, nidoviruses induce the formation of typical paired membranes and double-membrane structures that have commonly been referred to as “double-membrane vesicles” (DMVs) [25–28]. These structures are mainly found in the perinuclear area of the cell, where—according to immunofluorescence (IF) microscopy studies—de novo–made viral RNA and various replicase subunits colocalize, presumably in the viral RTC [16,17,28,29]. Immunoelectron microscopy (IEM) previously revealed that SARS-CoV nsp3 and nsp13 localize to the outside of DMVs and/or the region between DMVs. Although these proteins also colocalized in part with endoplasmic reticulum (ER) marker proteins [26,28,30], the origin of DMV membranes has remained undecided since other studies have implicated other organelles in the formation of RTCs and DMVs, e.g., late endosomes, autophagosomes, and most recently, the early secretory pathway and potentially also mitochondria [31–35]. Previous ultrastructural studies may have been hampered by the technical challenge of DMV preservation [28]. In particular, the DMV inner structure is fragile, and loss or collapse of DMV contents likely was a complicating factor. Although the use of cryofixation methods dramatically improved DMV preservation [28], our understanding of the three-dimensional (3-D) organization and origin of DMVs was hampered by the inherent limitations of analyzing “conventional” thin sections (100 nm) by electron microscopy (EM), in particular since the diameter of DMVs was estimated to be between 200 and 350 nm [28].

To develop a 3-D ultrastructural model for the RTC-related membrane alterations in SARS-CoV–infected cells, we have now employed electron tomography (ET; for reviews, see [36,37]). This technique uses a set of two-dimensional (2-D) transmission EM images, recorded at different specimen tilt angles with respect to the primary beam, for calculating a 3-D image (tomogram). Typically, the specimen is tilted over a range of ±65° in small tilt increments (1°), and an image is recorded at each tilt angle. The tomograms of infected cells allowed us to trace DMV membranes and establish previously unnoticed structural connections. In particular, ET revealed that coronavirus DMVs are not isolated vesicles, but instead are integrated into a unique reticulovesicular network of modified ER membranes, which also includes convoluted membranes that were not previously implicated in viral RNA synthesis. Strikingly, the latter structure—and not the DMVs—were primarily immunolabeled using antibodies recognizing viral replicase subunits. In contrast, immunolabeling with an antibody recognizing (presumably viral) dsRNA abundantly labeled the DMV interior. Since we could not discern a connection between the DMV interior and cytosol, our analysis raises several questions about the mechanism of DMV formation and the actual site of SARS-CoV RNA synthesis. The virus-induced “replication network” documented here places the early stages of the viral lifecycle and accompanying virus–host interactions in a new perspective.

## Results

### SARS-Coronavirus Infection Induces Multiple Distinct Membrane Alterations

Previously, we experienced that, compared to more traditional chemical fixation protocols, the preservation of the fragile coronavirus DMV structures could be significantly improved by using a combination of cryofixation and freeze substitution (FS) [28]. We now further refined the FS protocol, in particular by improving membrane contrast by adding 10% water to the FS medium [38].

**Figure 2:**
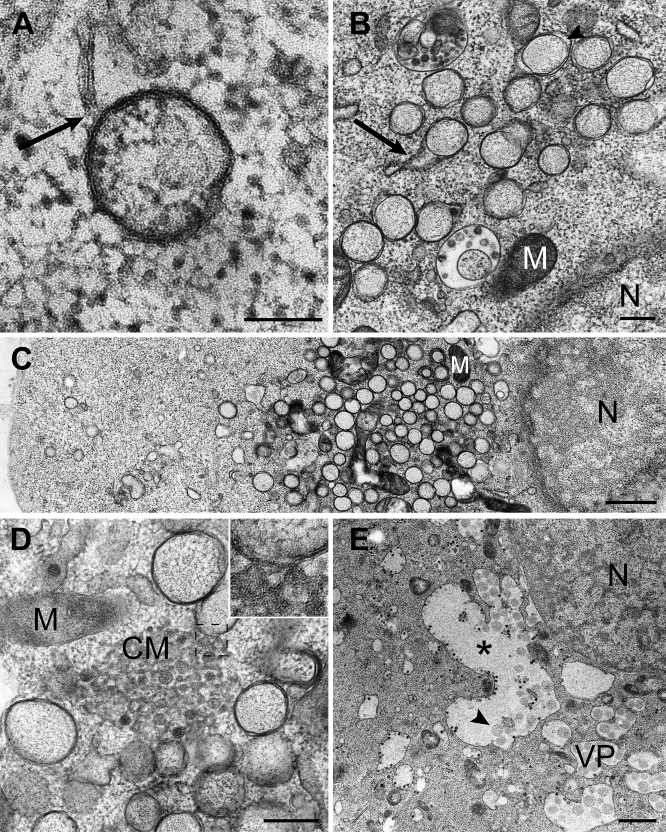
Overview of Membrane Structures Induced by SARS-CoV Infection Electron micrographs of SARS-CoV–infected Vero E6 cells. The cells were cryofixed and freeze substituted at 2 h p.i. (A), 8 h p.i. (B–D), or 10 h p.i. (E). (A) Early DMV as observed in a few sections, showing a connection (arrow) to a reticular membrane. (B) From 4 h p.i. on, clusters of DMVs began to form. Occasionally, connections between DMV outer membranes and reticular membrane structures were observed (arrow). Locally, luminal spacing between the DMV outer and inner membranes could be discerned (arrowhead). (C) As infection progressed, DMVs were concentrated in the perinuclear area (nucleus; N), often with mitochondria (M) lying in between. (D) Example of a cluster of CM, which were often surrounded by groups of DMVs. The structure seems to be continuous with the DMV outer membrane (inset). (E) During the later stages of infection, DMVs appeared to merge into VPs, which developed into large cytoplasmic vacuoles (asterisk) that contained not only single-membrane vesicles (arrowhead pointing to an example), but also (budding) virus particles. Scale bars represent 100 nm (A), 250 nm (B and D), or 1 μm (C and E).

Using these optimized conditions to prepare thin sections (100 nm) of SARS-CoV–infected Vero E6 cells, we could detect the first DMVs at 2 h postinfection (h p.i.) and were able to monitor the subsequent development of virus-induced membrane alterations. Early DMVs had sizes ranging from 150 to 300 nm, were distributed throughout the cytoplasm, and were sometimes located in the proximity of small reticular membranes with which, occasionally, they appeared to be connected (Figure 2A). From 4 h p.i. on, the number of DMVs increased dramatically, and DMV clusters were observed throughout the cell, again frequently accompanied by and sometimes clearly connected to reticular membrane structures (Figure 2B, arrow). As infection progressed, DMVs became increasingly concentrated in the perinuclear area of the cell (Figure 2C), in accordance with the available IF microscopy data for various SARS-CoV replicase subunits [16,28,29]. At 7 h p.i., a 100-nm-thick slice through the center of an infected Vero E6 cell generally contained between 200 and 300 DMVs. Initially, the DMV inner and outer membranes were generally tightly apposed, but occasionally, some luminal space between the two lipid bilayers could be discerned (Figure 2B, arrowhead). Although similar observations were previously made for different nidoviruses using a variety of chemical and cryofixation protocols, and despite the generally excellent preservation of cellular membranes, the documented fragility of coronavirus DMVs makes it clear that we cannot formally exclude the possibility that these local separations could result from preparation damage.

From 3 h p.i. on, we also observed large assemblies of convoluted membranes (CM), often in close proximity to DMV clusters (Figure 2D). These structures, with diameters ranging from 0.2 to 2 μm, are probably identical to the “reticular inclusions” that were first observed in cells infected with mouse hepatitis coronavirus (MHV) more than 40 y ago [39] and were later referred to as ‘clusters of tubular cisternal elements,' which may have a connection to the ER-Golgi intermediate compartment (ERGIC) [21]. We noticed that the SARS-CoV–induced CM resembled one of the replication-related membrane alterations induced by flaviviruses, which were proposed to be the site of viral genome translation and polyprotein processing [3,40,41]. In some of our images, the SARS-CoV–induced CM appeared to be continuous with both DMV outer membranes (Figure 2D; inset) and ER cisternae, suggesting a link to the viral RTC also in coronaviruses.

Especially at later stages of SARS-CoV infection (generally beyond 7 h p.i.), we observed packets of single-membrane vesicles surrounded by a common outer membrane, as previously described by Goldsmith et al. [27]. The diameter of these vesicle packets (VPs) ranged from 1 to 5 μm, and they sometimes included more than 25 inner vesicles (Figure 2E). In terms of size, morphology, electron density, and immunolabeling properties (see below), the vesicles contained in VPs strongly resembled the inner vesicles of DMVs, as seen at earlier time points. During these later stages of infection, the clustered single DMVs (Figure 2C) gradually disappeared, suggesting their merger into the VPs. The average outer diameter of DMV inner vesicles at 4 h p.i. was 250 ± 50 nm (*n* = 99), whereas later in infection, their average diameter (DMVs and VPs combined) increased to about 300 nm (310 ± 50 nm at 7 h p.i., 300 ± 50 μm at 10 h p.i.).

Our observations define VPs as a third distinct modification of intracellular membranes that is induced by SARS-CoV infection. By 10 h p.i., VPs appeared to have merged into even larger cytoplasmic vacuoles, containing both vesicles as well as significant numbers of budding and completed virions (Figure 2E). DMVs, CM, and VPs were not observed in mock-infected Vero E6 cells.

### Electron Tomography Reveals a Reticulovesicular Network of Modified ER Membranes in SARS-CoV–Infected Cells

**Figure 3:**
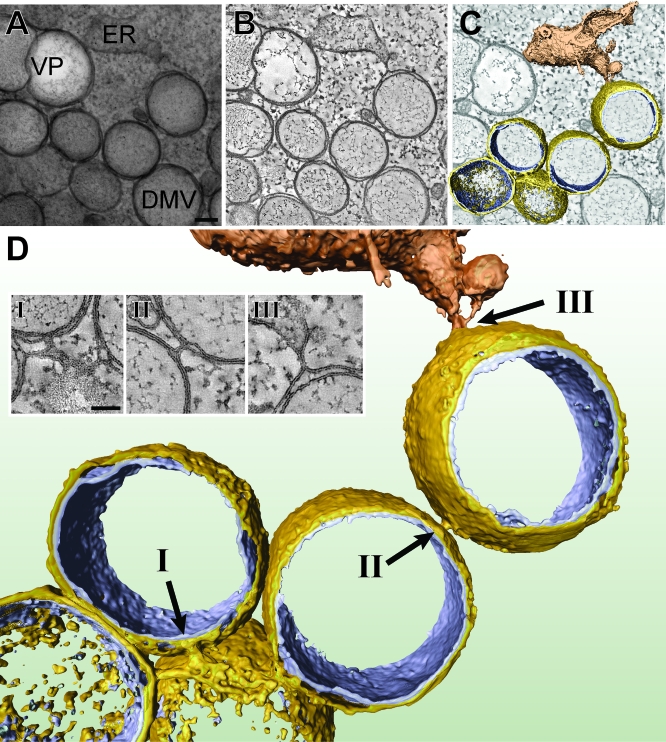
Electron Tomography Revealing the Interconnected Nature of SARS-CoV–Induced DMVs The series of images at the top illustrates how a 3-D surface-rendered model was derived by applying ET on a semi-thick section of a SARS-CoV–infected Vero E6 cell cryofixed at 7 h p.i. (A) A 0°-tilt transmission EM image of a 200-nm-thick resin-embedded section showing ER and a cluster of DMVs. The 10-nm gold particles were layered on top of the sections and were used as fiducial markers during subsequent image alignment. Scale bar represents 100 nm. (B) Using the IMOD software package (see Materials and Methods), tomograms were computed from dual-axis tilt series of the 200-nm-thick section shown in (A) (see also Videos S1 and S2). The tomographic slice shown here has a thickness of 1.2 nm. (C) The improved image from (B) following anisotropic diffusion filtering. The optimized signal-to-noise ratio facilitates thresholding and DMV surface rendering. See Figure S2 for a stereo image of this model. (D) Final 3-D surface-rendered model showing interconnected DMVs (outer membrane, gold; inner membrane, silver) and their connection to an ER stack (depicted in bronze). Arrows (I, II, and III) point to three clearly visible outer membrane continuities, with insets highlighting these connections in corresponding tomographic slices. Scale bar represents 50 nm.

**Figure 4:**
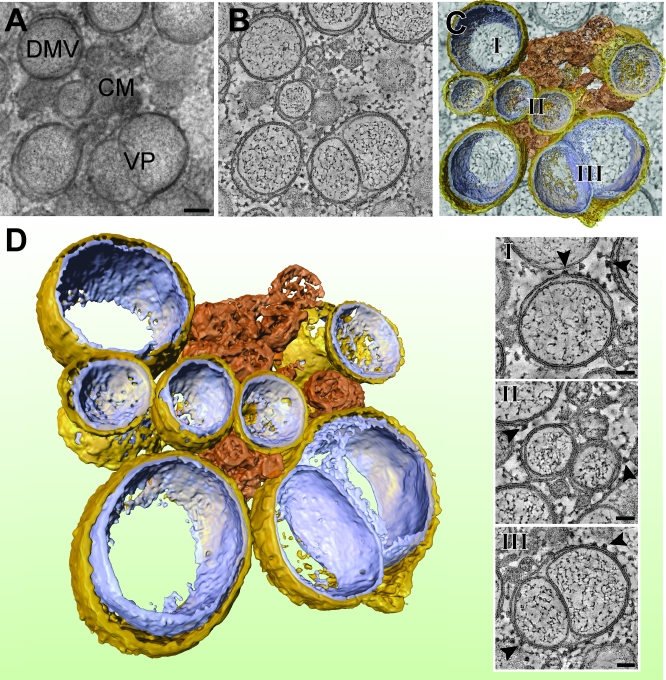
Electron Tomography of SARS-CoV–Induced CM, DMVs, and VPs As in Figure 3, (A–C) illustrate how a 3-D surface-rendered model was derived by applying ET on a semi-thick section of a SARS-CoV–infected Vero E6 cell cryofixed at 7 h p.i. Scale bar in (A) represents 100 nm. The type 1 VP present in this image shows an outer membrane that accommodates two tightly apposed inner vesicles with minimal luminal spacing. The insets (I, II, and III) below (C) show tomographic slices that highlight the presence of ribosomes (arrowheads) on DMV and VP outer membranes. Scale bar represents 50 nm. (D) shows the final 3-D surface-rendered model of this cluster of larger and smaller DMVs (outer membrane, gold; inner membrane, silver) of which the outer membranes are connected to the type 1 VP and a CM structure (depicted in bronze). See Figure S2 for a stereo image of this model.

**Figure 5:**
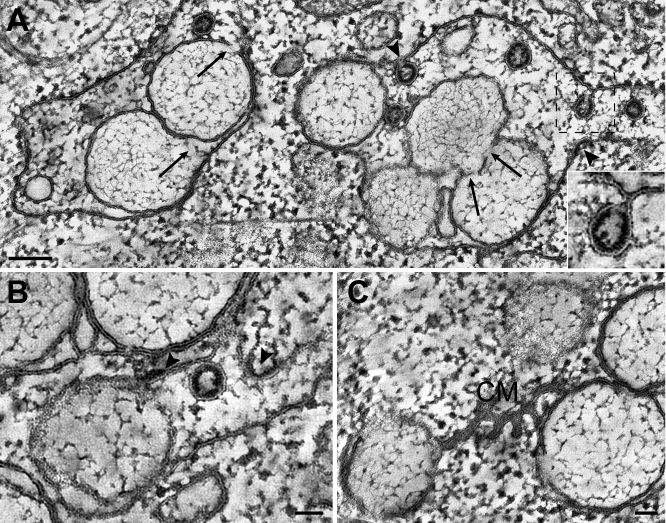
Electron Tomography of the SARS-CoV–Induced Reticulovesicular Membrane Network at a More Advanced Stage of Development Gallery of 10-nm-thick digital slices of tomograms (see legend to Figure 3B) from SARS-CoV–infected Vero E6 cells again cryofixed at 7 h p.i., but now selected for cells in which infection had progressed more than in others, allowing the visualization of more advanced stages of development of the virus-induced membrane alterations. (A) VP of the second type, showing a more relaxed outer membrane and several discontinuities (arrows) of inner vesicle membranes. New SARS-CoV particles can be seen budding from a VP outer membrane into the luminal space (arrowheads and inset; the inset shows a slightly tilted image to optimize the view). (B) Initial stage of virus budding from a VP outer membrane: formation of the electron-dense nucleocapsid structure at the cytosolic side of the membrane (arrowheads). (C) Example of a CM structure showing stacked membranes that are continuous with DMV outer membranes. Scale bars represent 100 nm (A) or 50 nm (B and C).

Although, occasionally, the analysis of “conventional” thin sections suggested CM and DMV outer membranes to be continuous and connected to ER cisternae, a more accurate assessment required an analysis in three dimensions. We therefore employed ET of semi-thick (200 nm) sections of cryofixed, SARS-CoV–infected Vero E6 cells. By using a specimen holder that could also be tilted around a second axis, perpendicular to both electron beam and first tilt axis, we obtained datasets, each consisting of 262 differently tilted 2-D images, which were used to produce a high-resolution reconstruction in three dimensions. Such “dual-axis” tomograms allowed us to visualize and analyze membrane continuities between the respective structures defined in the previous paragraph (as illustrated by Videos S1–S4 and Figures 3–5). The analysis was performed at 7 h p.i., a time point at which the various membrane alterations were all abundantly present in the absence of advanced cytopathology. Nevertheless, in some cells, infection had progressed more than in others, allowing the visualization of both advanced and earlier stages of infection in the same specimen.

Two major conclusions from this ET analysis were (1) that most or—likely—all coronavirus DMVs are interconnected by their outer membrane and (2) that they are part of an elaborate network that is continuous with the rough ER. As illustrated by the 3-D reconstruction in Figure 3, for most DMVs, we observed one or multiple thin (∼8 nm in diameter), “neck-like” connections of their outer membrane to the outer membranes of other DMVs, to CM, and to cisternae of the rough ER (Figure 3; insets). For example, in the two tomograms used for Videos S1 and S3, at least one such connection was visible for 77 out of 81 DMVs analyzed, strongly suggesting that for the remaining DMVs, such outer membrane connections existed but fell outside the volume reconstructed using these particular tomograms. Of the 77 DMVs for which at least one outer membrane connection was detected, 38 had a single connection, whereas 27, nine, and three DMVs had two, three, and four connections, respectively. Of these 131 connections, approximately one-half were between the outer membranes of DMVs and the other half were connections to ER or CM membranes, a ratio that was more or less stable when DMVs were differentiated in groups having one, two, or three connections. Consequently, the original concept of “free floating” coronavirus-induced DMVs (i.e., structures surrounded by two, fully detached unit membranes) should be adjusted, and it would appear more appropriate to describe DMVs as single-membrane vesicles confined in the lumen of an ER-connected membrane network. The VPs (Figures 4 and 5) and the tightly apposed membranes of the CM (Figure 5C) were found to be integral parts of the same reticulovesicular network. The ET analysis further suggested the presence of fibrous material inside DMV inner vesicles (Figures 3–5). Although ribosomes were clearly visible on rough ER cisternae and DMV/VP outer membranes (Figure 4, arrowheads; Video S1), they were not detected on the membranes or in the interior space of the inner vesicles.

By 7 h p.i., in part of the cells, the formation of VPs had begun (Figures 4 and 5), for which we could distinguish two different morphologies in our tomograms. In the first type (Figure 4; Video S3), the membranes of the adjacent inner vesicles were tightly apposed but intact, and there was little luminal space between the inner vesicles and the surrounding outer membrane. In contrast, the outer membrane of the second type of VP appeared more relaxed and generally contained multiple inner vesicles (Figure 5A; Video S4). Strikingly, instead of the intact inner membranes observed in DMVs and the first type of VP, we observed inner membrane discontinuities for many of the vesicles present in the second type of VP (Figure 5A), de facto resulting in the fusion of vesicles or in apparent connections with the lumen of the membrane compartment. Interestingly, we also observed virus budding from the outer membranes of the second type of VPs (Figure 5A and 5B, arrowheads), suggesting the ultimate convergence of RTC-associated membrane structures with compartments involved in virus assembly.

### SARS-CoV Replicase Subunits Localize Predominantly to Convoluted Membranes

**Figure 6:**
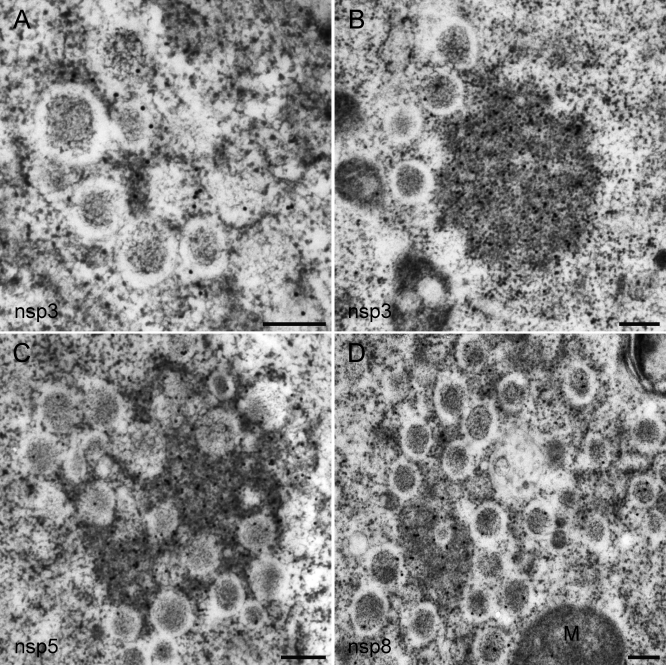
Immunogold EM of the SARS-CoV Replicase in Infected Cells SARS-CoV–infected Vero E6 cells were cryofixed at 8 h p.i. and processed for FS and IEM using rabbit antisera (see Materials and Methods). In all images, 15-nm colloidal gold particles conjugated to protein A were used for detection of primary antibodies. (A and B) Labeling for SARS-CoV nsp3 was mainly found on the electron-dense areas between DMVs, presumably representing CM as most clearly visible in (B). (C) Immunolabeling for SARS-CoV nsp5 (the viral main protease), which was essentially similar to that for nsp3. (D) When using an antiserum recognizing SARS-CoV nsp8 (the putative viral primase), the majority of label was again present on CM. However, a small fraction of the nsp8 signal was reproducibly found on the interior of DMVs. Scale bars represent 250 nm.

In order to assess the association of replicase subunits with the various coronavirus-induced membrane structures, we performed IEM experiments on SARS-CoV–infected Vero E6 cells. In view of previously experienced problems to preserve DMV ultrastructure for IEM [28], the FS protocol was further optimized, and samples were embedded in Lowicryl HM20. When using this fixation and embedding protocol, several of our antisera unfortunately no longer recognized their target, restricting our analysis—for the moment—to a relatively small number of replicase subunits. On the other hand, the various SARS-CoV–induced membrane alterations documented in the previous paragraphs could now readily be recognized in IEM samples (Figure 6). Furthermore, DMV inner structure was preserved, which had proven impossible in previous IEM studies [28].

For samples fixed at 8 h pi, highly specific immunogold labeling results were obtained with antisera [28] recognizing the large nsp3 subunit, which contains one of the viral proteases and is also a presumed transmembrane protein [23,42], the viral main protease nsp5 [43], and the nsp8 putative RNA primase, which has been postulated to be a subunit of the core RdRp complex [44,45]. Protein contrast was enhanced in these FS samples, due to the absence of stained membranes, revealing electron-dense areas between DMVs that were strikingly similar, both in size and localization, to the CM structures documented above (Figure 6). Remarkably, using all three reactive SARS-CoV antisera, CM were the most abundantly labeled structures. For nsp3 and nsp5, small numbers of gold particles were also found on DMV membranes, but the interior of DMVs (and VPs) was essentially devoid of label (Figure 6). In the case of nsp8, some labeling of the DMV interior was observed, but again the majority of the label localized between DMVs on the CM structures. In combination with our data from previous IF studies, documenting the colocalization of several key replicative enzymes [28], our IEM data suggest that the CM structures are the major site of SARS-CoV nsp accumulation.

### The Interior of Coronavirus-Induced DMVs Labels Abundantly for Double-Stranded RNA

A critical step in the replication of +RNA viruses is the production of a negative-stranded copy of the genome, which is used as a template for genome replication by the viral RdRp. Coronaviruses also generate a set of subgenome-length negative-strand RNAs, which serve as templates for subgenomic mRNA synthesis [19,20]. It is widely assumed that viral negative-strand RNA synthesis leads to the formation of partially and/or completely dsRNA structures, commonly referred to as replicative intermediates (RIs) and replicative forms (RFs) and, in the case of coronavirus subgenomic mRNA production, transcriptive intermediates (TIs) and transcriptive forms (TFs) [46,47]. Whereas RFs/TFs are (nearly) completely double stranded, and may accumulate, e.g., when RNA synthesis ceases and the last positive strand is not released from the negative strand, RIs/TIs are viewed as dynamic multistranded intermediates engaged in positive strand synthesis. They are thought to be only partially double stranded and contain multiple tails of nascent single-stranded RNA produced by the successive RdRp complexes engaged in copying the negative-strand template (see [47] and references therein).

**Figure 7:**
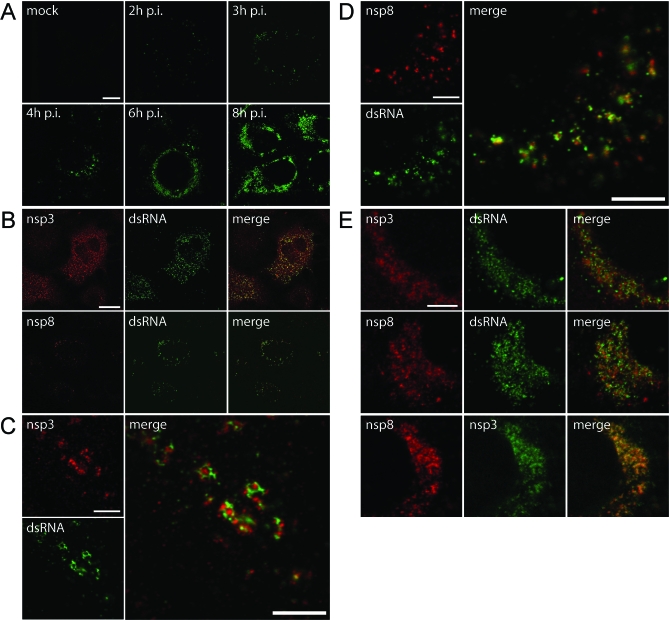
Detection of dsRNA in SARS-CoV–Infected Cells SARS-CoV–infected Vero E6 cells were fixed at various time points after infection and processed for IF assays using rabbit antisera recognizing different replicase subunits and a mouse monoclonal antibody specific for dsRNA. Imaging was done using a confocal laser scanning microscope. (A) Time-course experiment showing the development of dsRNA signal, which could be detected as early as 2 h p.i. Later in infection, the initially punctate cytoplasmic staining developed into a number of densely labeled areas close to the nucleus. (B) Dual-labeling IF assays using antisera recognizing dsRNA and either nsp3 or nsp8. The early signals for dsRNA and both nsps (here shown at 3 h p.i.) were found in close proximity of each other and partially overlapped. (C) High-resolution images of dual-labeling experiments for nsp3 and dsRNA early in infection (4 h p.i.), with the enlarged merged image illustrating that these signals were largely separated. (D) See (C), but now a dual-labeling experiment for nsp8 and dsRNA was performed. (E) High-resolution images of dual-labeling experiments for nsp3, nsp8, and dsRNA later in infection (6 h p.i.). Whereas the two nsps colocalized to a large extent (bottom row), this was less obvious when the labeling for dsRNA and replicase subunits was compared. Scale bars represent 10 μm (A), 25 μm (B), or 5 μm (C–E).

For a variety of +RNA viruses, the (presumed) dsRNA intermediates of replication have been visualized in situ by using antibodies recognizing dsRNA [40,48–50]. In particular, monoclonal antibody J2 [51], recognizing RNA duplexes larger than 40 base pairs, was reported to be a useful tool in recent IF studies [49,50]. We here used the J2 antibody in IF and EM studies, resulting in a highly specific labeling of SARS-CoV–infected cells, whereas mock-infected cells were essentially devoid of signal (Figure 7A). Even before immunodetection of nsps was feasible, the first IF signal for dsRNA could already be detected (at 2–3 h p.i.) as small but very bright foci throughout the cell (Figure 7A). By 4 h p.i., the distribution of dsRNA-containing foci generally mirrored that of nsp3, nsp5 (unpublished data), and nsp8 (Figure 7B). However, high-resolution confocal microscopy (Figure 7C and 7D) revealed that the overlap was far from complete, and frequently, multiple dsRNA foci appeared to surround an area that labeled for replicase. Later in infection, the labeling for both dsRNA and nsps was mainly concentrated in the perinuclear region (Figure 7E). Whereas different nsps colocalized to a large extent (Figure 7E, bottom row), this was less obvious when the labeling for dsRNA and replicase subunits was compared.

**Figure 8:**
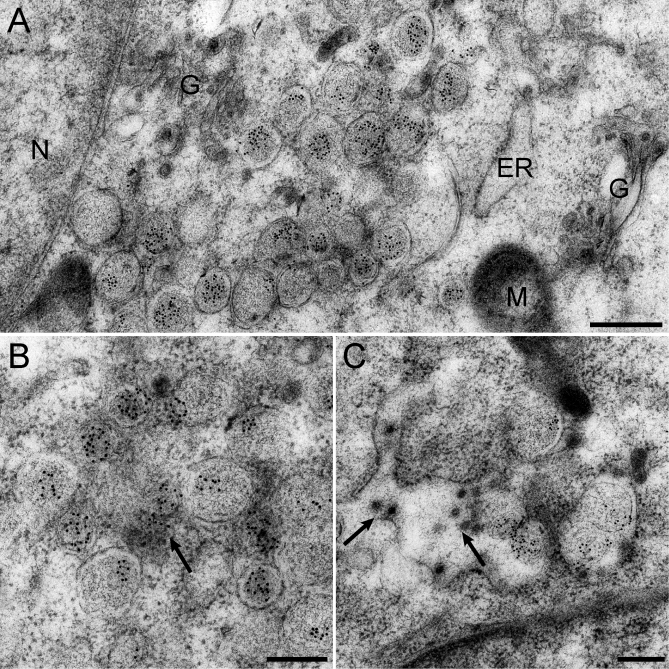
Immunogold EM Reveals Abundant dsRNA Labeling on the Interior of SARS-CoV–Induced DMVs SARS-CoV–infected Vero E6 cells were high-pressure frozen and processed for FS and IEM using a monoclonal antibody specific for dsRNA. In all images, 10-nm gold particles conjugated to protein A were used for detection of primary antibodies. (A) Overview of a SARS-CoV–infected cell at 7 h p.i., documenting the specificity of the dsRNA labeling and the abundant amount of label present on DMVs. G, Golgi complex; N, nucleus; M, mitochondria. (B) Cluster of abundantly labeled DMVs with additional labeling present in the area between the vesicles (arrow). (C) Type 2 VP showing abundant labeling for dsRNA on the interior of the inner vesicles. In addition, newly assembled virus particles can be seen in the lumen of the compartment (arrows). Scale bars represent 500 nm (A) or 250 nm (B and C).

In subsequent IEM experiments, the J2 antibody was found to retain its reactivity for dsRNA in sections of cells that had been embedded in Lowicryl, following the FS procedure described above. An abundant and highly specific labeling for dsRNA was observed on the interior of SARS-CoV–induced DMVs (Figure 8), with some additional label being present in the vicinity of DMVs where CM were frequently observed during our studies (Figure 8B). Also, type 1 and type 2 VPs were positive for dsRNA (Figure 8C), whereas (budding) virions present in these structures were always negative. Thus, our data revealed the accumulation of dsRNA, presumably of viral origin (see Discussion), in the interior vesicles of DMVs and VPs, and also suggested that the fibrous material observed in our ET analysis (Figures 3–5 and Videos S1 and S3) may consist (in part) of viral nucleic acids.

## Discussion

### Hijacking Cellular Membranes to Facilitate Coronavirus RNA Synthesis

The functional dissection of the multienzyme complexes that drive +RNA virus replication and transcription is essential for our understanding of the molecular biology of this important group of pathogens. Presumably, the membrane structures used to compartmentalize RTCs provide a suitable scaffold for viral RNA synthesis facilitate the organization of the viral replication cycle, and aid in evading or delaying antiviral host cell responses, including those that can be triggered by viral dsRNA [2–4,33,52,53]. The “replication structures” induced in cells infected with +RNA viruses can range from distinct spherular membrane invaginations to elaborate networks of CM and single- or double-membrane vesicles (for a recent review, see [5]). The first 3-D ultrastructural analysis of a replication structure of the spherular type was recently reported by Kopek et al. [7]. In an ET-based study, the replicase and RNA synthesis of flock house virus were found to be confined to spherular invaginations of the mitochondrial outer membrane. This “viral mini-organelle” was reported to be connected to the cytosol by a neck-like channel with a diameter of about 10 nm. This connection is assumed to be both sufficient and essential for the import of, e.g., nucleotides into replication spherules and for export of viral RNA products, which need to be released into the cytosol for translation and packaging.

**Figure 9:**
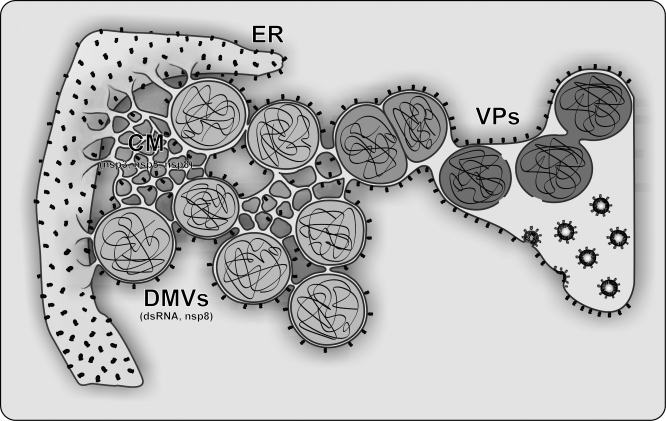
Electron Tomography-Based Model of the Network of Modified ER Membranes That Supports SARS-CoV RNA Synthesis A model showing the SARS-CoV–induced reticulovesicular network of modified membranes with which both viral replicase subunits and dsRNA are associated. Time postinfection increases from left to right. The various interconnected membrane structures documented in this study are depicted. The CM, the outer membranes of DMVs and VPs, and—ultimately—membrane compartments used for virus budding were all found to be continuous with the rough ER, as underlined by the presence of ribosomes on each of these components. DMV inner membranes and the interior of the vesicles, which contained as yet undefined “fibrous material,” were devoid of ribosomes but labeled abundantly for dsRNA. Ultimately, the network appears to connect membrane structures involved in SARS-CoV RNA synthesis to sites at which the assembly of new virions occurs and may thus contribute to the organization of successive stages in the viral life cycle in both time and space.

We have now employed ET to analyze the replication structures of SARS-CoV, a prominent member of the coronavirus group which—at about 7,000 amino acids—encodes the largest known +RNA virus replicase [14]. DMVs had previously been observed in cells infected with coronaviruses and related nidoviruses [25–28,30], and the precise origin of their membranes had remained debated. They were generally assumed to be “free floating” vesicles associated with viral RNA synthesis. However, our present ET analysis has revealed that they form a unique reticulovesicular membrane network (Figure 9) with which both viral replicase subunits and dsRNA are associated (Figures 6–8). The network is continuous with the rough ER and contains in its lumen numerous “inner vesicles,” which stand out for their relatively large size (200–300-nm diameter), their number (several hundred, possibly more than 1,000 vesicles per cell), and for the fact that they label abundantly for dsRNA. Remarkably, however, their interior does not appear to be connected to the cytosol (see also below). The fact that the average diameter of the SARS-CoV–induced DMVs (250–300 nm) exceeds the maximal thickness (∼200 nm) of the sections that could be used for ET made it generally impossible to visualize their entire perimeter. However, the number of inter-DMV connections that could be observed in the reconstructed volume (at least one for 95% of the DMVs analyzed, with more than half of those having multiple connections) justifies the conclusion that they form an integrated network and makes it highly unlikely that free DMVs exist (Figures 3–5). VPs and CM structures are also an integral part of the network (Figures 3–5 and 9), and in particular, the latter structures appear to be a major site of immunolabeling for SARS-CoV nsps. Essentially similar observations were made for cells infected with a second coronavirus, MHV (unpublished data). Our studies identify the ER as the source for a virus-induced membrane network that integrates CM, DMVs, and VPs, although the (additional) involvement of the ERGIC remains a possibility [21]. In combination with biochemical studies, the ultrastructural description of this network is important to take our understanding of coronavirus RTC structure and function to the next level.

Finally, our analysis of cells at more advanced stages of SARS-CoV infection (Figure 5) opens the intriguing possibility that the membrane network involved in virus replication is continuous (or merges) with membranes involved in virus assembly. For MHV, based on IF microscopy studies using the nsp13 helicase and viral membrane (M) protein as markers for RTCs and virus assembly sites [21], respectively, such a connection was previously proposed [54], but could not be corroborated in our studies using the same protein markers in SARS-CoV–infected cells [28]. According to the data presented in this study, the bulk of the labeling for nsps is found on the CM structures (Figure 6), not on DMVs, which would explain the minimal overlap between the nsp labeling and that for the M protein [28]. Furthermore, it cannot be excluded that merger of type 2 VPs and compartments involved in virus budding is a relatively rare event that could result from general cytopathology and/or fusion of different membrane compartments. Notably, in some of the larger VPs (e.g., see Figure 2E), a kind of polarity was observed, with budding and mature virions mostly on one side and the inner vesicles of (former) DMVs on the other, as if two previously “dedicated compartments” recently merged into a larger vesicle. Although the juxtaposition and functional connection of compartments involved in genome replication, encapsidation, and assembly remains a fascinating idea, a thorough quantitative analysis of SARS-CoV assembly is beyond the scope of this paper and would require the collection of extensive datasets, in particular around the peak time of virus assembly (10–12 h p.i.).

### The Enigma of the Double Membrane

By analogy with the replication-associated “membrane spherules” of several other +RNA viruses [7,55], it was anticipated that the DMV interior would be connected to the cytosol, thus allowing import of (macro)molecules required for RNA synthesis and export of RNA products, e.g., for translation and packaging. However, our tomograms revealed a sealed DMV inner membrane and an uninterrupted outer membrane that was clearly continuous with other membrane structures. The two tomograms that were the basis for Figures 3 and 4 and Videos S1 and S3 were scrutinized for discontinuities of DMV inner and/or outer membranes, with the expectation of finding at least one such connection per vesicle when DMV interior and cytosol would indeed be continuous. Neck-like connections between the outer membranes of different DMVs were readily discerned (see above; Figure 3), and the quality of our images also allowed the high-resolution visualization of, e.g., membrane necks of budding virions (Figure 5). However, for the vast majority of DMVs, an extensive search for a repetitive pattern showing a neck, channel, or other type of structure connecting DMV interior and cytosol remained negative. For only one out of 78 DMVs visible in Videos S1 and S3, an aligned gap of both inner and outer membrane could be detected (Figure S1A). Furthermore, in three other DMV profiles, the inner membrane was locally disrupted (Figure S1B–S1D), but since these sites also showed local separation of the two leaflets of the bilayer, we consider it likely that these interruptions were fixation or processing artifacts. Given the previously documented fragility of the DMV inner membrane in particular, this would not be surprising, and this property may also be related to the puzzling inner membrane discontinuities observed for type 2 VPs late in infection (Figure 5 and Video S4). However, for the vast majority of DMVs in our images, the inner membrane was found to be uninterrupted, and thus, the DMV interior appears not connected to the cytosol.

In view of the resolution provided by our tomograms, we are confident that we would have readily detected connections to the cytosol with a diameter (8–10 nm) in the range previously described for other +RNA virus replication structures [7,55]. Thus, our data suggest that—at least at the moment of fixation—DMV inner membranes form closed vesicles and their morphogenesis has now become one of the major unresolved issues. It should be stressed that we cannot exclude the possibility that proteinaceous pores or transporters may be present in DMV membranes, since similar complexes (e.g., the translocon) have not been recognized in situ in EM/ET studies yet. However, despite the fact that the large coronavirus proteome was recently found to include several unexpected and unprecedented functions, proposing the existence of such a channel would seem highly speculative at this moment. Three coronavirus replicase subunits (nsp3, nsp4, and nsp6) contain hydrophobic domains that are each predicted to traverse the membrane multiple times [23,24,56]. Their properties have not been characterized in detail, but the recent phenotypic characterization of a temperature-sensitive MHV mutant with a lesion in nsp4 revealed a dramatic reduction of DMV formation at the restrictive temperature, thus clearly implicating this protein in the formation of the reticulovesicular network documented in this study [34]. Still, apart from the question whether the transmembrane nsps are able to form membrane-spanning channels or recruit host proteins capable of forming such a connection, other conceptual problems would remain. For example, the alignment of the channels spanning the inner and outer membranes would appear to be a requirement, much like it has been proposed for the sophisticated TOM and TIM complexes engaged in import across mitochondrial outer and inner membranes [57,58]. Moreover, the transport across membranes of a large, negatively charged RNA molecule like the approximately 30-kb coronavirus genome poses a challenge that in biology appears to be met only by the nuclear pore complex.

In addition to the recent data on MHV nsp4 [34], results obtained with the distantly related arteriviruses indicate that the (predicted) membrane-spanning nsps of nidoviruses are likely to play a critical role in inducing membrane alterations. It was shown that the expression of two such arterivirus nsps sufficed to induce paired membranes and DMVs similar to those found upon virus infection [25,59,60]. Most likely, these subunits are first inserted into “regular” ER membranes, which may thus also be the site of early viral RNA synthesis. When replication leads to a rapid increase of replicase expression, the accumulating transmembrane nsps may induce membrane pairing and curvature, due to, e.g., their specific structural features, oligomerization, or recruitment of cellular factors involved in membrane bending. The notion that inner and outer bilayer may be “physically associated,” due, for example, to interacting luminal domains of protein partners present in the two membranes, is supported by the fact that the two membranes remain tightly associated just up to the point where narrow neck-like connections protrude to the outer membrane of other vesicles or compartments (Figure 3). Apparently, at later time points after infection when DMVs merge into the larger vesicle packets, the inner membranes are able to more and more detach from the outer membrane. Interestingly, during our recent (unpublished) studies using the drug brefeldin A, which interferes with vesicular transport and de facto results in fusion of Golgi complex and ER into one large, dilated compartment, similar observations could be made much earlier in infection. This would suggest that the interaction between the two membranes eventually weakens, possibly in particular when the outer membrane network becomes dilated due to cytopathology and/or merger of multiple vesicles.

Presumably, membrane pairing is followed by the wrapping of membrane cisternae around cytosolic constituents and leads to the membrane fission event that is needed to explain the sealed DMV inner membrane. However, despite the presence of several hundred DMVs in infected cells and despite the extensive EM analysis of hundreds of cells in the course of this study, we were unable to find morphological profiles that seemed obvious examples of an actual DMV-forming fission event. Although some smaller DMVs were sometimes observed (Video S3), the average dimensions of their inner compartments (200–300 nm in diameter) should have made the detection of nascent DMV structures straightforward. Arguably, DMV formation might be very rapid, and thus rarely captured, or obscured in, e.g., the complex architecture of the CM structure, where smaller DMVs were sometimes apparent (Figure 4 and Video S3). Alternatively, the conspicuous absence of ribosomes from DMV inner membranes lends some credibility to a scenario involving a preformed inner vesicle derived from another membrane source.

The observed narrow neck-like connections in the network (Figure 3) and the fact that many DMVs were found to have multiple (up to four) of such outer membrane connections with other DMVs, CM, or ER also leaves the possibility that additional fusion and fission events may occur during the formation or maturation of the network, which would obviously hamper the analysis of the initial DMV forming event. The future identification of inhibitory drugs or dominant-negative mutants of viral or host proteins involved in this step may facilitate the visualization of this crucial intermediate stage in DMV morphogenesis.

### Comparison with Other +RNA Virus Replication Complexes

In infected cells, several other groups of +RNA viruses induce membrane alterations that differ from the spherular membrane invaginations described, e.g., for nodaviruses [7] and alphaviruses [55]. In the case of picornaviruses (for a recent review, see [5]), the pioneering work of the Bienz laboratory demonstrated that poliovirus RNA replication occurs on the cytosolic surface of ER-derived vesicles [61], which aggregate into rosette-like structures [1]. However, the first detectable negative-stranded RNA of poliovirus is associated with regular ER cisternae, which may thus be the initial site of RNA synthesis [62]. Other studies revealed that poliovirus-induced vesicles may have a double membrane [63] and implicated the autophagic pathway in their formation [64]. A similar hypothesis was launched to explain MHV DMV formation [32]. Despite a convincing link between overall MHV replication and the expression of a host protein with a critical function in autophagy (Apg5), the “autophagy hypothesis” was contradicted by IF studies using autophagosomal marker proteins [28,30].

Our studies may in fact have uncovered a closer parallel to the membranes with which the replication complexes of flaviviruses are associated. Ultrastructural studies of cells infected with Kunjin virus have defined various characteristic membrane structures, which were implicated in viral RNA synthesis on the basis of immunolabeling and biochemical studies ([40]; for reviews, see [3,5]). These structures include “convoluted membranes” and “vesicle packets,” terms that we have chosen to adopt in our study, without wanting to imply a direct ultrastructural or functional similarity. Whereas the flavivirus CM have been implicated in replicase polyprotein synthesis and processing, the VPs were proposed to be the site of viral RNA synthesis, in particular because they could be immunolabeled for replicase subunits, dsRNA, and de novo–synthesized viral RNA that had been metabolically labeled by bromouridine (BrU) incorporation [3]. A key premise, however, in the current model proposed for flaviviruses [3,65,66] is the idea that—as in the case of viruses employing spherular replication compartments (see above; [7])—the interior of the vesicles enclosed in the VPs are connected to the cytosol.

For the DMVs induced by coronaviruses and other nidoviruses, a similar hypothesis was among the previously formulated models [25], but—as explained above—in our SARS-CoV tomograms, an open connection between DMV interior and cytosol could not be discerned. Recent biochemical studies on the in vitro activity of SARS-CoV RTCs, which were associated with membrane fractions prepared from infected cells, revealed that a detergent treatment is required to render the viral RNA synthesizing complex susceptible to digestion with proteases or nucleases [67]. Thus, the isolated RTC appears to be protected by at least one membrane, a conclusion also drawn from similar biochemical studies on flavivirus RTCs, leading to an alternative model [68] in which flavivirus VPs would be “topologically similar” to coronavirus VPs and consist of a closed inner vesicle surrounded by an outer membrane that is continuous with CM and ER. If a future ET analysis of flavivirus replication structures were to confirm this similarity, we would essentially be faced with the same question for both virus groups [68]: if RNA synthesis would indeed occur inside closed DMVs or VPs, how then are import and export across the double membrane achieved?

### Pinpointing the Active Site of SARS-CoV RNA Synthesis

The presence of both viral nsps and dsRNA on the SARS-CoV–induced membrane network strongly suggests its involvement in viral replication and transcription. However, the apparent separation in immunolabeling studies (Figures 6–8) between the bulk of the nsps and most of the dsRNA emphasizes the need to pinpoint the active coronavirus RTC. In particular the exact role in viral RNA synthesis of the DMV inner vesicle, its “fibrous content,” and its abundant labeling for dsRNA are intriguing. Extensive proteolytic processing of replicase polyproteins pp1a and pp1ab (Figure 1) is assumed to be a critical posttranslational step in the activation of coronavirus replicative enzyme functions. It is also a complicating factor in immunolabeling studies since antibodies will commonly recognize both mature cleavage products and larger processing intermediates. Moreover, immunolabeling will merely reveal the site of accumulation of specific antigens, not necessarily their site of synthesis.

The fact that most of the label for SARS-CoV nsps was present on CM may seem incompatible with the presence of most of the dsRNA signal on DMVs. If, however, as proposed for flaviviruses, the coronavirus CM would be the site of polyprotein synthesis and processing, abundant labeling of this region could be expected, in particular for the two viral proteases, nsp3 and nsp5, that were detected on the CM in this study. The labeling observed for the putative nsp8 primase differed slightly, with some label consistently being present on the DMV interior (Figure 6D). The nsp8 subunit possesses a secondary RNA polymerase activity and has been postulated to be part of the core enzyme complex of the virus [44,45]. Additional antisera, in particular targeting the viral key enzymes encoded in ORF1b (Figure 1), are currently being generated to increase our possibilities for detection of subunits of the multicomponent SARS-CoV RTC. Also, the search for suitable antibodies against cellular marker proteins continues, which could aid in defining the interaction with the host cell's secretory pathway in more detail.

As recently concluded for hepatitis C virus [65], a huge excess of nonstructural proteins may be produced in virus-infected cells, with only a fraction of these molecules actively participating in viral RNA synthesis at any point in time. Likewise, the labeling for dsRNA, although widely considered a marker for +RNA virus RTCs [3,49,50], does not formally pinpoint RTC activity. Clearly, molecules inside active RTCs may be among the dsRNA strands recognized, as is strongly suggested by colocalization of dsRNA and newly made viral RNA following BrU pulse labeling [69]. On the other hand, however, it is likely that part of the signal, a part that may in fact vary between different viruses, represents dsRNA molecules that are no longer actively engaged in viral RNA synthesis. In this context, it is noteworthy that the calculations on the number of active replication complexes in hepatitis C virus replicon cell lines (less than 100; [65]) are not easily reconciled with the much larger number of discrete foci detected in such cells when labeling with the J2 anti-dsRNA monoclonal antibody [50]. Given these considerations, it would be most straightforward to localize the site of activity of the SARS-CoV RTC early in infection, using ultrastructural studies that are combined with pulse labeling of viral RNA synthesis using BrU [69] or radioisotope-labeled nucleosides [70], or by transfecting the corresponding nucleoside triphosphates. Experiments to explore whether it is technically feasible to combine such an approach with the cryo-EM and FS fixation protocols required for SARS-CoV DMV preservation are in progress. In our opinion, previous IEM studies using BrU labeling of MHV-infected cells [26] cannot be considered conclusive in view of the obvious loss during fixation of the DMV inner vesicles, and possibly also the CM. Nevertheless, the BrU labeling detected by these authors on DMV outer membranes and surrounding structures suggests that at least part of the newly made RNA was cytosolic after a 1-h labeling interval.

In conclusion, a scenario in which part, or even most, of the SARS-CoV dsRNA signal represents molecules that are not present in active RTCs (Figures 7 and 8) cannot be ruled out at present. In this alternative scenario, the active complex might, for example, localize to the CM, where small amounts of dsRNA labeling and the bulk of the viral nsps were detected. The subsequent formation of DMVs could then even be postulated to constitute an elegant mechanism to conceal viral RNA and aid in the evasion of dsRNA-triggered antiviral host responses. A variety of recent studies have made clear that coronaviruses are capable of interacting and interfering with the innate immune system at multiple levels, likely also depending on the cell type involved (for a recent review, see [71]). Both SARS-CoV and MHV [72–74] were found to counteract the induction of interferon via cytoplasmic pattern recognition receptors that can sense the presence of viral dsRNA [9,10] and possibly also viral negative-strand RNAs carrying uncapped 5′-triphosphates [75]. Further analysis of the structure, interactions, and function of the coronavirus RTC may reveal to which extent this property should be attributed to the unusual network of modified membranes with which coronavirus RNA synthesis appears to be associated.

## Materials and Methods

### Virus, cells, and antisera.

SARS-CoV strain Frankfurt-1 (kindly provided by Dr. H. F. Rabenau and Dr. H. W. Doerr [Johann-Wolfgang-Goethe-Universität, Frankfurt am Main, Germany]; [15]) was used to infect Vero E6 cells. All work with live SARS-CoV was performed inside biosafety cabinets in the biosafety level 3 facility at Leiden University Medical Center. A multiplicity of infection of 10 was used in all experiments, and infection rates were routinely confirmed in IF assays. A panel of rabbit antisera against the SARS-CoV replicase, including the nsp3, nsp5, and nsp8 subunits, was described previously [28]. A mouse monoclonal antibody J2 [51], which is specific for dsRNA, was purchased from Scicons.

### Electron microscopy.

For ultrastructural morphological investigations, SARS-CoV–infected Vero E6 cells were prefixed (for biosafety reasons) overnight with 3% paraformaldehyde in 0.1 M PHEM buffer (60 mM piperazide-1,4-bis[2-ethanesulfonic acid], 25 mM HEPES, 2 mM MgCl_2_, 10 mM EGTA) at various time points after infection. For cryofixation, cell monolayers adhered to Thermanox coverslips (Nunc) were plunged into liquid ethane. Freeze substitution was performed at −90 °C in an automated freeze-substitution system (Leica) using an FS medium consisting of 90% acetone and 10% water, containing 1% osmium tetroxide and 0.5 % uranyl acetate. After washing with pure acetone at room temperature, the samples were embedded in epoxy LX-12 resin. Thin sections were contrasted with uranyl acetate and lead hydroxide, and subsequently viewed at 80 kV with a Philips CM-10 transmission electron microscope.

For IEM, infected cell monolayers were cryofixed by either plunging them into liquid ethane or by high-pressure freezing using a Leica EM PACT2. The freeze substitution was performed using anhydrous acetone containing 0.25% glutaraldehyde and 0.1% uranyl acetate. After washing with ethanol, samples were infiltrated with Lowicryl HM20 and polymerized under UV light at −50 °C. Thin sections were labeled with specific antisera [28], which were detected with protein A-gold particles (10 or 15 nm). A bridging rabbit–anti-mouse IgG antibody (DakoCytomation) was used for mouse monoclonal antibodies. Grids were contrasted with uranyl acetate and lead hydroxide, and subsequently viewed with a Philips CM-10 transmission electron microscope.

When quantifying DMVs per infected cell, thin sections were cut in the direction parallel to the substrate, and the slice producing the largest nuclear diameter was analyzed, since this plane was generally found to contain the largest number of DMVs. Electron micrographs (between 20 and 100) covering the entire cross-section of the cell were recorded, and to facilitate counting, these were digitally merged to produce a single image representing a 100-nm-thick plane through the center of the infected cell. Merged images were analyzed with Zoomify software. Only DMVs for which the surrounding bilayers could be readily distinguished were counted, and their diameter was measured using ImageJ software (http://rsb.info.nih.gov/ij/).

### Electron tomography.

Freeze-substituted infected cell samples, processed for morphological investigation as described above, were used to cut 200-nm-thick sections. To facilitate the image alignment that is required for the subsequent image reconstruction step, a suspension of 10-nm gold particles was layered on top of the sections as fiducial markers. For dual-axis tomography, two single-axis tilt series were recorded of the specimens with an FEI T12 transmission electron microscope operating at an acceleration voltage of 120 kV. Per single-axis tilt series, 131 images were recorded at 1° tilt increments between −65 °C and 65 °C. Automated tomography acquisition software was used (Xplore 3D; FEI Company). Images were acquired with a cooled slow-scan charge-coupled device (CCD) camera (4k Eagle; FEI Company) with 4,096 × 4,096 pixels and were recorded by binning 2. The electron microscope magnification was 18,500×, corresponding to a pixel size of 1.2 nm at the specimen level. To enable dual-axis tomography, the specimens were rotated 90° around the *z*-axis using a dual-axis tilt tomography holder (Fishione; model 2040). To compute the electron tomogram, the dual-axis tilt series were aligned by means of the fiducial markers using the IMOD software package [76]. The size of the voxels in the tomograms corresponds to 1.2 nm. Full datasets have been deposited in the Cell Centered Database (http://ccdb.ucsd.edu; [77]) under accession numbers 6020–6023, respectively, containing the datasets of the tomograms shown in Videos S1, S3, and S4, and a Zoomify image showing a high-resolution cross-section of an entire SARS-CoV–infected cell.

The 3-D surface-rendered reconstructions of viral structures and adjacent cellular features were processed using AMIRA Visualization Package (TSG Europe) by surface rendering and thresholding. During this process, some volumes were denoised using the nonlinear anisotropic diffusion filtering [78]. Denoised volumes were used only for producing the surface-rendered masks. Final analyses and representations were done using undenoised data (either masked or unmasked).

### Immunofluorescence microscopy.

Infected cells on glass coverslips were fixed with 3% paraformaldehyde in PBS at various time points after infection and were processed for IF microscopy essentially as described previously [79]. Following permeabilization, single- or dual-labeling IF assays were carried out with rabbit antisera and/or mouse monoclonal antibodies, which were detected using indocarbocyanine (Cy3)-conjugated donkey anti-rabbit immunoglobulin (Ig) and Alexa Fluor 488-conjugated goat anti-mouse Ig secondary antibodies, respectively (Molecular Probes). For dual-labeling experiments with two rabbit antisera recognizing different SARS-CoV nonstructural proteins, the anti-nsp3 antibodies were directly coupled to Alexa Fluor 488, as described previously [28].

Samples were examined with a Zeiss Axioskop 2 fluorescence microscope (equipped with the appropriate filter sets, a digital Axiocam HRc camera, and Zeiss Axiovision 4.2 software) (Carl Zeis, Microimaging) or with a Leica SP5 confocal laser scanning microscope, using a pinhole size of 1 airy unit (for both channels) to give optical sections with a theoretical thickness of 236 nm. Images were minimally optimized for contrast and brightness using Adobe Photoshop CS2.

## Supporting Information

Figure S1 In-Depth Analysis of Discontinuities in the Membranes of SARS-CoV–Induced DMVs See the legend to Figure 3 for details. A total of 78 DMVs in the two tomograms that were the basis for Figure 3A and 3B and Videos S1 and S2 were scrutinized for discontinuities of DMV inner and/or outer membranes that might reveal a connection between the DMV interior and the cytoplasm. However, an extensive search for a repetitive pattern showing a neck, channel, or other type of structure connecting the DMV interior and cytoplasm remained negative. One out of 78 DMVs ([A]; arrow) showed a small, aligned gap of both inner and outer membrane. In three other DMV profiles ([B–D]; arrows), the inner membrane was locally disrupted, but the separation of the two leaflets of the bilayer made it likely that these discontinuities were artifacts that had occurred during fixation and processing of the fragile DMV inner structure. The scale bar represents 50 nm.
(2.15 MB JPG)

Figure S2 Stereo Images of the 3-D Surface-Rendered Models Presented in Figures 3C and 4D Anaglyph images were produced and superimposed to provide a stereoscopic 3-D effect when viewed with spectacles with red (left) and green (right) glasses.
(6.68 MB JPG)

Video S1 Animation through a *z*-Series of 1-nm-Thick Digital Slices (Total Depth 200 nm) of a Dual-Axis Electron Tomogram of a SARS-CoV–Infected Vero E6 Cell at 7 h p.i. The video shows a group of interconnected DMVs and also shows the connections of DMV outer membranes with the ER. The tightly apposed double membranes and fibrous material inside the DMVs are clearly visible. To facilitate image alignment during image reconstruction, a suspension of 10-nm gold particles was layered on top of the sections as fiducial markers.
(7.25 MB WMV)

Video S2 Animation Illustrating the Derivation of the Model Presented in Figure 3D from the Dual-Axis Tomogram of a 200-nm-Thick Section of a SARS-CoV–Infected Vero E6 Cell, as Shown in Video S1
(8.25 MB WMV)

Video S3 Animation through a *z*-Series of 1-nm-Thick Digital Slices (Total Depth 200 nm) of a Dual-Axis Electron Tomogram of a SARS-CoV-Infected Vero E6 Cell at 7 h p.i. The video shows a group of interconnected DMVs and also a type 1 VP. The tightly apposed double membranes and fibrous material inside the DMVs are clearly visible. To facilitate image alignment during image reconstruction, a suspension of 10-nm gold particles was layered on top of the sections as fiducial markers.
(3.99 MB WMV)

Video S4 Animation through a *z*-Series of 1-nm-Thick Digital Slices (Total Depth 200 nm) of a Dual-Axis Electron Tomogram of a SARS-CoV–Infected Vero E6 Cell at 7 h p.i. The video illustrates a relatively late stage of SARS-CoV–induced membrane alterations, during which DMVs seem to merge into larger VPs (type 2). Also, various stages of virus budding can be observed at the outer membranes of these type 2 VPs. Note that clear discontinuities in the membranes of the inner vesicles can be observed, ostensibly resulting in fusion of DMV contents with each other and with the lumen of the VP. To facilitate image alignment during image reconstruction, a suspension of 10-nm gold particles was layered on top of the sections as fiducial markers.
(3.31 MB WMV)

## Abbreviations

3-D: three-dimensional
CM: convoluted membranes
DMV: double-membrane vesicle
dsRNA: double-stranded RNA
EM: electron microscopy
ER: endoplasmic reticulum
ERGIC: endoplasmic reticulum–Golgi intermediate compartment
ET: electron tomography
FS: freeze substitution
IEM: immunoelectron microscopy
IF: immunofluorescence
h p.i.: hours postinfection
MHV: mouse hepatitis virus
nsp: nonstructural protein
ORF: open reading frame
RdRp: RNA-depended RNA polymerase
+RNA: positive-strand RNA
RTC: replication/transcription complex
SARS: severe acute respiratory syndrome
SARS-CoV: severe acute respiratory syndrome-coronavirus
VP: vesicle packet

## References

[pbio-0060226-b001] Bienz K, Egger D, Pfister T, Troxler M (1992). Structural and functional characterization of the poliovirus replication complex. J Virol.

[pbio-0060226-b002] Ahlquist P (2006). Parallels among positive-strand RNA viruses, reverse-transcribing viruses and double-stranded RNA viruses. Nat Rev Microbiol.

[pbio-0060226-b003] Mackenzie J (2005). Wrapping things up about virus RNA replication. Traffic.

[pbio-0060226-b004] Novoa RR, Calderita G, Arranz R, Fontana J, Granzow H, Risco C (2005). Virus factories: associations of cell organelles for viral replication and morphogenesis. Biol Cell.

[pbio-0060226-b005] Netherton C, Moffat K, Brooks E, Wileman T (2007). A guide to viral inclusions, membrane rearrangements, factories, and viroplasm produced during virus replication. Adv Virus Res.

[pbio-0060226-b006] Kirkegaard K, Jackson WT (2005). Topology of double-membraned vesicles and the opportunity for non-lytic release of cytoplasm. Autophagy.

[pbio-0060226-b007] Kopek BG, Perkins G, Miller DJ, Ellisman MH, Ahlquist P (2007). Three-dimensional analysis of a viral RNA replication complex reveals a virus-induced mini-organelle. PLoS Biol.

[pbio-0060226-b008] Miller S, Krijnse Locker J (2008). Modification of intracellular membrane structures for virus replication. Nat Rev Microbiol.

[pbio-0060226-b009] Haller O, Kochs G, Weber F (2006). The interferon response circuit: induction and suppression by pathogenic viruses. Virology.

[pbio-0060226-b010] Garcia-Sastre A, Biron CA (2006). Type 1 interferons and the virus-host relationship: a lesson in detente. Science.

[pbio-0060226-b011] Peiris JSM, Guan Y, Yuen KY (2004). Severe acute respiratory syndrome. Nat Med.

[pbio-0060226-b012] Pyrc K, Berkhout B, van der Hoek L (2007). The novel human coronaviruses NL63 and HKU1. J Virol.

[pbio-0060226-b013] Ziebuhr J (2004). Molecular biology of severe acute respiratory syndrome coronavirus. Curr Opin Microbiol.

[pbio-0060226-b014] Gorbalenya AE, Enjuanes L, Ziebuhr J, Snijder EJ (2006). Nidovirales: evolving the largest RNA virus genome. Virus Res.

[pbio-0060226-b015] Thiel V, Ivanov KA, Putics A, Hertzig T, Schelle B (2003). Mechanisms and enzymes involved in SARS coronavirus genome expression. J Gen Virol.

[pbio-0060226-b016] Prentice E, McAuliffe J, Lu XT, Subbarao K, Denison MR (2004). Identification and characterization of severe acute respiratory syndrome coronavirus replicase proteins. J Virol.

[pbio-0060226-b017] Harcourt BH, Jukneliene D, Kanjanahaluethai A, Bechill J, Severson KM (2004). Identification of severe acute respiratory syndrome coronavirus replicase products and characterization of papain-like protease activity. J Virol.

[pbio-0060226-b018] Snijder EJ, Bredenbeek PJ, Dobbe JC, Thiel V, Ziebuhr J (2003). Unique and conserved features of genome and proteome of SARS-coronavirus, an early split-off from the coronavirus group 2 lineage. J Mol Biol.

[pbio-0060226-b019] Pasternak AO, Spaan WJM, Snijder EJ (2006). Nidovirus transcription: how to make sense…. J Gen Virol.

[pbio-0060226-b020] Sawicki SG, Sawicki DL, Siddell SG (2007). A contemporary view of coronavirus transcription. J Virol.

[pbio-0060226-b021] Krijnse Locker J, Ericsson M, Rottier PJM, Griffiths G (1994). Characterization of the budding compartment of mouse hepatitis virus: evidence that transport from RER to the Golgi complex requires only one vesicular transport step. J Cell Biol.

[pbio-0060226-b022] Masters PS (2006). The molecular biology of coronaviruses. Adv Virus Res.

[pbio-0060226-b023] Kanjanahaluethai A, Chen Z, Jukneliene D, Baker SC (2007). Membrane topology of murine coronavirus replicase nonstructural protein 3. Virology.

[pbio-0060226-b024] Oostra M, Te Lintelo EG, Deijs M, Verheije MH, Rottier PJ (2007). Localization and membrane topology of the coronavirus nonstructural protein 4: involvement of the early secretory pathway in replication. J Virol.

[pbio-0060226-b025] Pedersen KW, van der Meer Y, Roos N, Snijder EJ (1999). Open reading frame 1a-encoded subunits of the arterivirus replicase induce endoplasmic reticulum-derived double-membrane vesicles which carry the viral replication complex. J Virol.

[pbio-0060226-b026] Gosert R, Kanjanahaluethai A, Egger D, Bienz K, Baker SC (2002). RNA replication of mouse hepatitis virus takes place at double-membrane vesicles. J Virol.

[pbio-0060226-b027] Goldsmith CS, Tatti KM, Ksiazek TG, Rollin PE, Comer JA (2004). Ultrastructural characterization of SARS coronavirus. Emerging Infect Dis.

[pbio-0060226-b028] Snijder EJ, van der Meer Y, Zevenhoven-Dobbe J, Onderwater JJM, van der Meulen J (2006). Ultrastructure and origin of membrane vesicles associated with the severe acute respiratory syndrome coronavirus replication complex. J Virol.

[pbio-0060226-b029] Ivanov KA, Thiel V, Dobbe JC, van der Meer Y, Snijder EJ (2004). Multiple enzymatic activities associated with severe acute respiratory syndrome coronavirus helicase. J Virol.

[pbio-0060226-b030] Stertz S, Reichelt M, Spiegel M, Kuri T, Martínez-Sobrido L (2007). The intracellular sites of early replication and budding of SARS-coronavirus. Virology.

[pbio-0060226-b031] van der Meer Y, Snijder EJ, Dobbe JC, Schleich S, Denison MR (1999). Localization of mouse hepatitis virus nonstructural proteins and RNA synthesis indicates a role for late endosomes in viral replication. J Virol.

[pbio-0060226-b032] Prentice E, Jerome WG, Yoshimori T, Mizushima N, Denison MR (2004). Coronavirus replication complex formation utilizes components of cellular autophagy. J Biol Chem.

[pbio-0060226-b033] Kirkegaard K, Taylor MP, Jackson WT (2004). Cellular autophagy: surrender, avoidance and subversion by microorganisms. Nat Rev Microbiol.

[pbio-0060226-b034] Clementz MA, Kanjanahaluethai A, O'Brien TE, Baker SC (2008). Mutation in murine coronavirus replication protein nsp4 alters assembly of double membrane vesicles. Virology.

[pbio-0060226-b035] Verheije MH, Raaben M, Mari M, Te Lintelo EG, Reggiori F (2008). Mouse hepatitis coronavirus RNA replication depends on GBF1-mediated ARF1 activation. PLoS Pathog.

[pbio-0060226-b036] Koster AJ, Grimm R, Typke D, Hegerl R, Stoschek A (1997). Perspectives of molecular and cellular electron tomography. J Struct Biol.

[pbio-0060226-b037] Frey TG, Perkins GA, Ellisman MH (2006). Electron tomography of membrane-bound cellular organelles. Annu Rev Biophys Biomol Struct.

[pbio-0060226-b038] Walther P, Ziegler A (2002). Freeze substitution of high-pressure frozen samples: the visibility of biological membranes is improved when the substitution medium contains water. J Microsc.

[pbio-0060226-b039] David-Ferreira JF, Manaker RA (1965). An electron microscope study of the development of a mouse hepatitis virus in tissue culture cells. J Cell Biol.

[pbio-0060226-b040] Westaway EG, Mackenzie JM, Kenney MT, Jones MK, Khromykh AA (1997). Ultrastructure of Kunjin virus-infected cells: colocalization of NS1 and NS3 with double-stranded RNA, and of NS2b with NS3, in virus-induced membrane structures. J Virol.

[pbio-0060226-b041] Mackenzie JM, Jones MK, Westaway EG (1999). Markers for trans-Golgi membranes and the intermediate compartment localize to induced membranes with distinct replication functions in flavivirus-infected cells. J Virol.

[pbio-0060226-b042] Neuman BW, Joseph JS, Saikatendu KS, Serrano P, Chatterjee A (2008). Proteomics analysis unravels the functional repertoire of coronavirus nonstructural protein 3. J Virol.

[pbio-0060226-b043] Ziebuhr J, Snijder EJ, Gorbalenya AE (2000). Virus-encoded proteinases and proteolytic processing in the Nidovirales. J Gen Virol.

[pbio-0060226-b044] Zhai Y, Sun F, Li X, Pang H, Xu X3 (2005). Insights into SARS-CoV transcription and replication from the structure of the nsp7-nsp8 hexadecamer. Nat Struct Mol Biol.

[pbio-0060226-b045] Imbert I, Guillemot JC, Bourhis JM, Bussetta C, Coutard B (2006). A second, non-canonical RNA-dependent RNA polymerase in SARS coronavirus. EMBO J.

[pbio-0060226-b046] Sethna PB, Hung SL, Brian DA (1989). Coronavirus subgenomic minus-strand RNAs and the potential for mRNA replicons. Proc Natl Acad Sci U S A.

[pbio-0060226-b047] Sawicki D, Wang T, Sawicki S (2001). The RNA structures engaged in replication and transcription of the A59 strain of mouse hepatitis virus. J Gen Virol.

[pbio-0060226-b048] Stollar BD, Stollar V (1970). Immunofluorescent demonstration of double-stranded RNA in the cytoplasm of Sindbis virus-infected cells. Virology.

[pbio-0060226-b049] Weber F, Wagner V, Rasmussen SB, Hartmann R, Paludan SR (2006). Double-stranded RNA is produced by positive-strand RNA viruses and DNA viruses but not in detectable amounts by negative-strand RNA viruses. J Virol.

[pbio-0060226-b050] Targett-Adams P, Boulant S, McLauchlan J (2007). Visualization of double-stranded RNA in cells supporting hepatitis C virus RNA replication. J Virol.

[pbio-0060226-b051] Schonborn J, Oberstrass J, Breyel E, Tittgen J, Schumacher J (1991). Monoclonal antibodies to double-stranded RNA as probes of RNA structure in crude nucleic acid extracts. Nucleic Acids Res.

[pbio-0060226-b052] Salonen A, Ahola T, Kaariainen L (2004). Viral RNA replication in association with cellular membranes. Curr Top Microbiol Immunol.

[pbio-0060226-b053] Wileman T (2006). Aggresomes and autophagy generate sites for virus replication. Science.

[pbio-0060226-b054] Bost AG, Prentice E, Denison MR (2001). Mouse hepatitis virus replicase protein complexes are translocated to sites of M protein accumulation in the ERGIC at late times of infection. Virology.

[pbio-0060226-b055] Froshauer S, Kartenbeck J, Helenius A (1988). Alphavirus RNA replicase is located on the cytoplasmic surface of endosomes and lysosomes. J Cell Biol.

[pbio-0060226-b056] Sparks JS, Lu X, Denison MR (2007). Genetic analysis of murine hepatitis virus nsp4 in virus replication. J Virol.

[pbio-0060226-b057] Kutik S, Guiard B, Meyer HE, Wiedemann N, Pfanner N (2007). Cooperation of translocase complexes in mitochondrial protein import. J Cell Biol.

[pbio-0060226-b058] Endo T, Yamamoto H, Esaki M (2003). Functional cooperation and separation of translocators in protein import into mitochondria, the double-membrane bounded organelles. J Cell Sci.

[pbio-0060226-b059] Snijder EJ, van Tol H, Roos N, Pedersen KW (2001). Non-structural proteins 2 and 3 interact to modify host cell membranes during the formation of the arterivirus replication complex. J Gen Virol.

[pbio-0060226-b060] Posthuma CC, Pedersen KW, Lu Z, Joosten RG, Roos N (2008). Formation of the arterivirus replication/transcription complex: a key role for nonstructural protein 3 in the remodeling of intracellular membranes. J Virol.

[pbio-0060226-b061] Rust RC, Landmann L, Gosert R, Tang BL, Hong WJ (2001). Cellular COPII proteins are involved in production of the vesicles that form the poliovirus replication complex. J Virol.

[pbio-0060226-b062] Egger D, Bienz K (2005). Intracellular location and translocation of silent and active poliovirus replication complexes. J Gen Virol.

[pbio-0060226-b063] Schlegel A, Giddings THJ, Ladinsky MS, Kirkegaard K (1996). Cellular origin and ultrastructure of membranes induced during poliovirus infection. J Virol.

[pbio-0060226-b064] Jackson WT, Giddings TH, Taylor MP, Mulinyawe S, Rabinovitch M (2005). Subversion of cellular autophagosomal machinery by RNA viruses. PLoS Biol.

[pbio-0060226-b065] Quinkert D, Bartenschlager R, Lohmann V (2005). Quantitative analysis of the hepatitis C virus replication complex. J Virol.

[pbio-0060226-b066] Miyanari Y, Atsuzawa K, Usuda N, Watashi K, Hishiki T (2007). The lipid droplet is an important organelle for hepatitis C virus production. Nat Cell Biol.

[pbio-0060226-b067] van Hemert MJ, van den Worm SHE, Knoops K, Mommaas AM, Gorbalenya AE (2008). SARS-coronavirus replication/transcription complexes are membrane-enclosed and need a host factor for activity in vitro. PLoS Pathog.

[pbio-0060226-b068] Uchil PD, Satchidanandam V (2003). Architecture of the flaviviral replication complex. J Biol Chem.

[pbio-0060226-b069] Westaway EG, Khromykh AA, Mackenzie JM (1999). Nascent flavivirus RNA colocalized in situ with double-stranded RNA in stable replication complexes. Virology.

[pbio-0060226-b070] Bienz K, Egger D, Pasamontes L (1987). Association of polioviral proteins of the P2 genomic region with the viral replication complex and virus-induced membrane synthesis as visualized by electron microscopic immunocytochemistry and autoradiography. Virology.

[pbio-0060226-b071] Versteeg GA, Spaan WJM, Perlman S, Gallagher T, Snijder EJ (2008). Host cell responses to coronavirus infections. Nidoviruses.

[pbio-0060226-b072] Versteeg GA, Bredenbeek PJ, van den Worm SH, Spaan WJ (2007). Group 2 coronaviruses prevent immediate early interferon induction by protection of viral RNA from host cell recognition. Virology.

[pbio-0060226-b073] Zhou H, Perlman S (2007). Mouse hepatitis virus does not induce Beta interferon synthesis and does not inhibit its induction by double-stranded RNA. J Virol.

[pbio-0060226-b074] Roth-Cross JK, Martinez-Sobrido L, Scott EP, Garcia-Sastre A, Weiss SR (2007). Inhibition of the alpha/beta interferon response by mouse hepatitis virus at multiple levels. J Virol.

[pbio-0060226-b075] Hornung V, Ellegast J, Kim S, Brzozka K, Jung A (2006). 5′-Triphosphate RNA is the ligand for RIG-I. Science.

[pbio-0060226-b076] Kremer JR, Mastronarde DN, McIntosh JR (1996). Computer visualization of three-dimensional image data using IMOD. J Struct Biol.

[pbio-0060226-b077] Martone ME, Tran J, Wong WW, Sargis J, Fong L (2008). The cell centered database project: an update on building community resources for managing and sharing 3D imaging data. J Struct Biol.

[pbio-0060226-b078] Frangakis AS, Hegerl R (2001). Noise reduction in electron tomographic reconstructions using nonlinear anisotropic diffusion. J Struct Biol.

[pbio-0060226-b079] van der Meer Y, van Tol H, Krijnse Locker J, Snijder EJ (1998). ORF1a-encoded replicase subunits are involved in the membrane association of the arterivirus replication complex. J Virol.

